# Activation of the Type VI Secretion System in the Squid Symbiont *Vibrio fischeri* Requires the Transcriptional Regulator TasR and the Structural Proteins TssM and TssA

**DOI:** 10.1128/JB.00399-21

**Published:** 2021-10-12

**Authors:** Stephanie Smith, Fernanda Salvato, Aditi Garikipati, Manuel Kleiner, Alecia N. Septer

**Affiliations:** a Department of Earth, Marine and Environmental Sciences, University of North Carolina, Chapel Hill, North Carolina, USA; b Department of Plant and Microbial Biology, North Carolina State University, Raleigh, North Carolina, USA; Geisel School of Medicine at Dartmouth

**Keywords:** type VI secretion systems, *Aliivibrio fischeri*, microbial interactions, genomic island, symbiosis

## Abstract

Bacteria have evolved diverse strategies to compete for a niche, including the type VI secretion system (T6SS), a contact-dependent killing mechanism. T6SSs are common in bacterial pathogens, commensals, and beneficial symbionts, where they affect the diversity and spatial structure of host-associated microbial communities. Although T6SS gene clusters are often located on genomic islands (GIs), which may be transferred as a unit, the regulatory strategies that promote gene expression once the T6SS genes are transferred into a new cell are not known. We used the squid symbiont Vibrio fischeri to identify essential regulatory factors that control expression of a strain-specific T6SS encoded on a GI. We found that a transcriptional reporter for this T6SS is active only in strains that contain the T6SS-encoding GI, suggesting the GI encodes at least one essential regulator. A transposon screen identified seven mutants that could not activate the reporter. These mutations mapped exclusively to three genes on the T6SS-containing GI that encode two essential structural proteins (a TssA-like protein and TssM) and a transcriptional regulator (TasR). Using T6SS reporters, reverse transcription-PCR (RT-PCR), competition assays, and differential proteomics, we found that all three genes are required for expression of many T6SS components, except for the TssA-like protein and TssM, which are constitutively expressed. Based on these findings, we propose a model whereby T6SS expression requires conserved structural proteins, in addition to the essential regulator TasR, and this ability to self-regulate may be a strategy to activate T6SS expression upon transfer of T6SS-encoding elements into a new bacterial host.

**IMPORTANCE** Interbacterial weapons like the T6SS are often located on mobile genetic elements, and their expression is highly regulated. We found that two conserved structural proteins are required for T6SS expression in Vibrio fischeri. These structural proteins also contain predicted GTPase and GTP binding domains, suggesting their role in promoting T6SS expression may involve sensing the energetic state of the cell. Such a mechanism would provide a direct link between T6SS activation and cellular energy levels, providing a “checkpoint” to ensure the cell has sufficient energy to build such a costly weapon. Because these regulatory factors are encoded within the T6SS gene cluster, they are predicted to move with the genetic element to activate T6SS expression in a new host cell.

## INTRODUCTION

Across diverse environments, bacterial populations engage in fierce competition for the limited space and resources that they require for survival and self-propagation. This competition exerts a strong selective pressure on microbes to engage in an arms race to evolve the most competitive mechanisms that incur the minimum cost. One example of a fast-evolving competitive mechanism is the type VI secretion system (T6SS). It has been predicted that T6SSs evolved from bacteriophages (1) and are often found within genomic islands (GIs) across diverse bacterial species (2). Indeed, T6SSs are encoded in the genomes of ∼25% of sequenced Gram-negative bacteria from a diversity of phyla and habitats. These bacteria are found free-living as well as associated with eukaryotic hosts, where they act as commensals (3–6), pathogens (7–20), or beneficial symbionts (21–24) for insects, plants, and animals across both terrestrial and marine environments (25). This broad distribution suggests the secretion system can confer an advantage to bacteria in diverse niches on a global scale.

The T6SS killing mechanism relies on a diverse pool of polymorphic effector/immunity proteins. In *Proteobacteria*, 13 core proteins comprise the secretion apparatus that injects effector proteins directly into competitor cells (26–32). If the competitor cell is exposed to toxic effectors for which it lacks immunity, the cell dies and lyses (33), thus allowing the space and resources that would have been consumed by its progeny to be used by the T6SS^+^ winner (33–35). These effector/immunity genes rapidly diversify among closely related strains and species, resulting in evolved bacterial populations that are unable to occupy the same space (27, 36, 37). Thus, T6SSs are capable of shaping the diversity and even spatial distribution of microbial populations.

Bioinformatics analysis of T6SSs indicates there are distinct evolutionary lineages of T6SSs that can be acquired and lost by diverse bacterial populations (38, 39). However, in order for these horizontally acquired T6SSs to be retained in new host genomes and passed on to progeny cells, they must confer an advantage to the cell that is maintained under positive selection. This presents a dilemma—how do newly acquired T6SSs activate expression of the secretion apparatus in order to confer a beneficial function and thus ensure the genes are not lost? One possibility is that, like phages (40–42), the T6SS-encoding gene clusters also encode a mechanism to self-activate. In theory, once the T6SS is expressed and confers a selective advantage to the bacterial population, its maintenance over longer time scales would allow for further integration of T6SS expression into the cellular network. Although past work has identified a large diversity of stimuli that lead to regulation of T6SSs in different bacteria for optimal use in their specific niche (19, 43–45), little is known about broadly conserved regulatory factors that may allow T6SS-encoding gene clusters to activate their own expression.

Here, we use the bioluminescent squid symbiont Vibrio fischeri as a model organism to identify essential broadly conserved regulators for T6SS gene expression. V. fischeri carries two predicted T6SS gene clusters: one cluster on chromosome I (T6SS1), which is found in all sequenced V. fischeri isolates, and a second cluster on chromosome II (T6SS2) (21). Here, we focus on T6SS2, which we previously showed is located within a strain-specific genomic island and is active both in culture and in its natural host, where it is required for spatial separation of competing genotypes in different crypts within the symbiotic light organ (21). In this study, we used reporters for T6SS2 transcription and random transposon mutagenesis to identify genetic factors that are essential for T6SS2 expression in culture. In addition, we examined how in *trans* expression of these proteins impacts the ability of V. fischeri to modulate T6SS2 gene expression and killing ability. Finally, we used a proteomics approach to obtain a systems-level view of how these essential factors control protein expression in the cell. This study identifies broadly conserved genetic factors that are required to activate T6SS expression, which may be a strategy for self-activation to promote the dissemination of T6SSs on mobile elements.

## RESULTS

### T6SS2 promoter reporter activity correlates with the presence of the T6SS2 GI.

**FIG 1:**
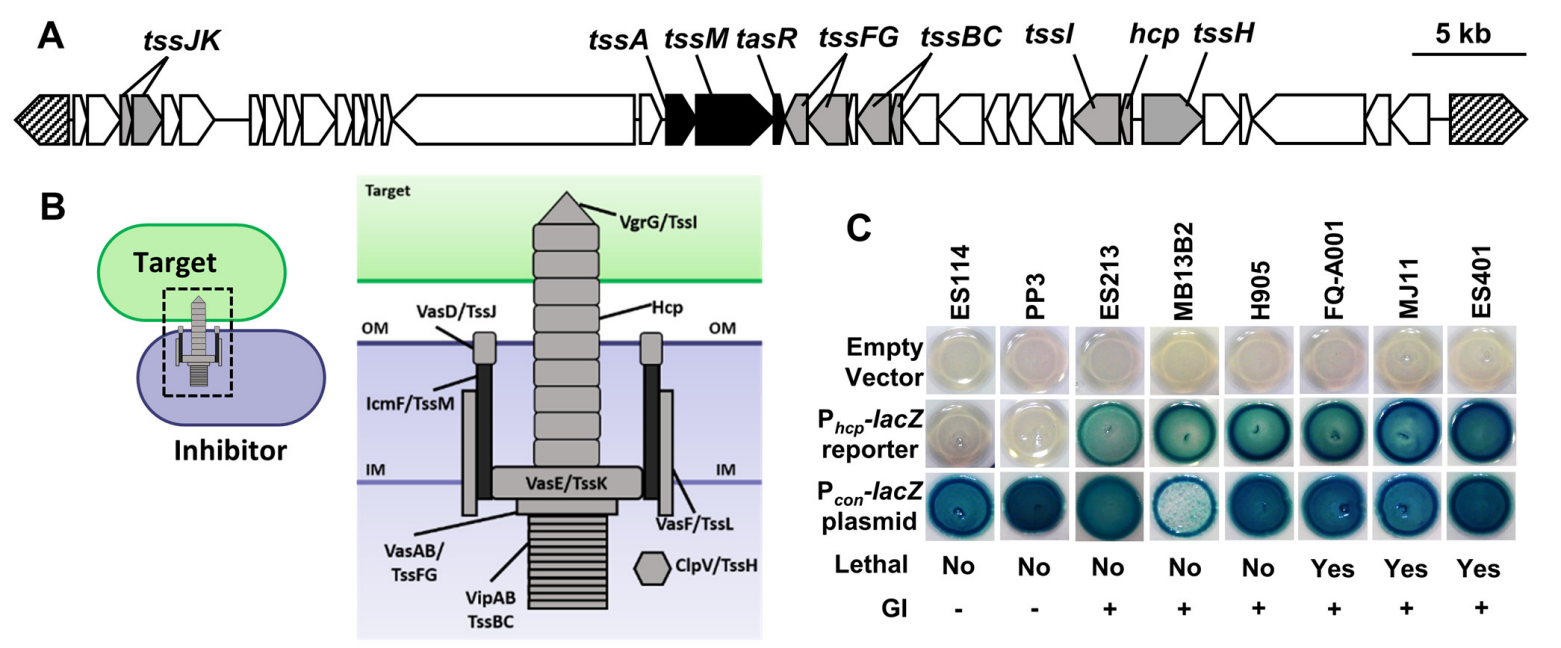
T6SS2 promoter activity is strain specific and correlates with the presence of the genomic island. (A) T6SS2 genomic island (GI) located on chromosome II of V. fischeri strain ES401 (lethal). ES401 carries an ∼50-kb gene cluster containing predicted T6SS genes on chromosome II (T6SS2) that is lacking in nonlethal strains. Regulatory genes identified in this study are indicated in black. Structural T6SS genes are indicated in gray, and hatched arrows indicate conserved genes flanking the ES401 genomic island. (B) Structural model of a T6SS inhibitor cell firing into a target cell based on V. cholerae and other systems. (C) P*_hcp_-lacZ* promoter reporter activity for 8 representative V. fischeri strains with P*_hcp_-lacZ* (pAG01 reporter), a promoterless *lacZ* (pAKD701 empty vector), or a vector containing a synthetic promoter to constitutively drive *lacZ* expression (P*_con_-lacZ*, pJLB207 constitutive vector). Lethality indicates ability to eliminate ES114 in a coincubation, as shown previously by Speare et al. (21). The presence of the GI was determined using PCR amplification (21). Images were taken after 24 h of incubation on LBS–X-Gal. Blue indicates increased P*_hcp_*-*lacZ* activity, and white indicates no P*_hcp_-lacZ* reporter activity. Experiments were performed at least four times (*n* = 12), and a representative experiment is shown.

Because diverse isolates of V. fischeri display a natural genetic variation regarding the presence or absence of the T6SS2-encoding GI, this species represents a unique opportunity to examine how the prevalence of a T6SS-encoding GI impacts T6SS regulation. We hypothesized that if the GI encodes a mechanism to self-activate, then we will see a strain-specific activation of a T6SS transcriptional reporter. We used the previously described *lacZ*-based promoter reporter for the *hcp_2* gene (44), which is the first of a putative 13-gene operon encoding several essential T6SS structural proteins (21) (Fig. 1A). The *hcp* gene encodes a hemolysin-coregulated protein (Hcp) that comprises the inner tube of the T6SS syringe (Fig. 1B) and is required for T6SS-mediated cytotoxicity across diverse species, including V. fischeri (11, 46, 47). We previously showed that *hcp_2* promoter reporter activity and expression of core T6SS proteins are correlated and regulated by conditions in the medium: reporter activity is low in cells grown in liquid culture where no killing is apparent and is activated in cells grown on surfaces or in hydrogel, where T6SS2 proteins are more abundant and T6SS-dependent killing is observed (44). Thus, the P*_hcp_* reporter is a good indicator of T6SS2 protein expression.

To determine whether strain genotype influences P*_hcp_* reporter activity, we moved the promoterless *lacZ* plasmid (empty vector control) and the P*_hcp_*-*lacZ* reporter plasmid into eight different V. fischeri strains. We grew the control and reporter strains on agar medium supplemented with 5-bromo-4-chloro-3-indolyl-β-d-galactopyranoside (X-Gal) to observe the relative reporter activity. Promoter activity can be assessed visually using this qualitative reporter assay because the LacZ enzyme cleaves the X-Gal substrate, resulting in a blue-pigmented product. Therefore, if a promoter is highly active, colonies appear dark blue, but if the promoter is less active, colonies appear white or light blue. All strains carrying the empty vector were white on X-Gal plates, indicating little to no *lacZ* expression in the promoterless control (Fig. 1C). We observed strain-specific differences in color for the P*_hcp_* reporter strains: two strains lacking the GI (ES114 and PP3) were white on X-Gal plates, and all six GI-containing strains (ES213, MB13B2, H905, FQ-A001, MJ11, and ES401) were blue (Fig. 1C), including isolates whose T6SS2 is naturally nonfunctional and therefore do not kill target cells (21). To ensure that this strain-specific outcome was due to regulation of the P*_hcp_* reporter and not a result of strain-specific differences in the ability to transport and/or metabolize the X-Gal substrate, we also used a plasmid in which a constitutive promoter (P*_con_*) drives *lacZ* expression (48). All strains containing the P*_con_-lacZ* plasmid appeared blue on X-Gal plates (Fig. 1C). Taken together, these data indicate that the strain-specific ability to activate the P*_hcp_*-*lacZ* reporter correlates with the presence of the T6SS2 genomic island, suggesting one or more genes on the genomic island are required for reporter activation.

### A random transposon screen reveals regulatory mutants.

To identify the essential regulators of P*_hcp_* in an unbiased way, we performed random transposon mutagenesis on the ES401 reporter strain. If the transposon disrupts a gene that is required to activate P*_hcp_*-*lacZ*, then the mutant reporter colony will appear white on X-Gal plates. Our initial screen of reporter activity revealed that strains containing a nonfunctional T6SS2 are still able to activate the reporter, indicating that a functional T6SS2 is not required for reporter activity (Fig. 1C). Therefore, we expected a transposon mutant screen to reveal genes specifically involved in regulation of our promoter reporter, rather than proteins required only for general T6SS function.

**FIG 2:**
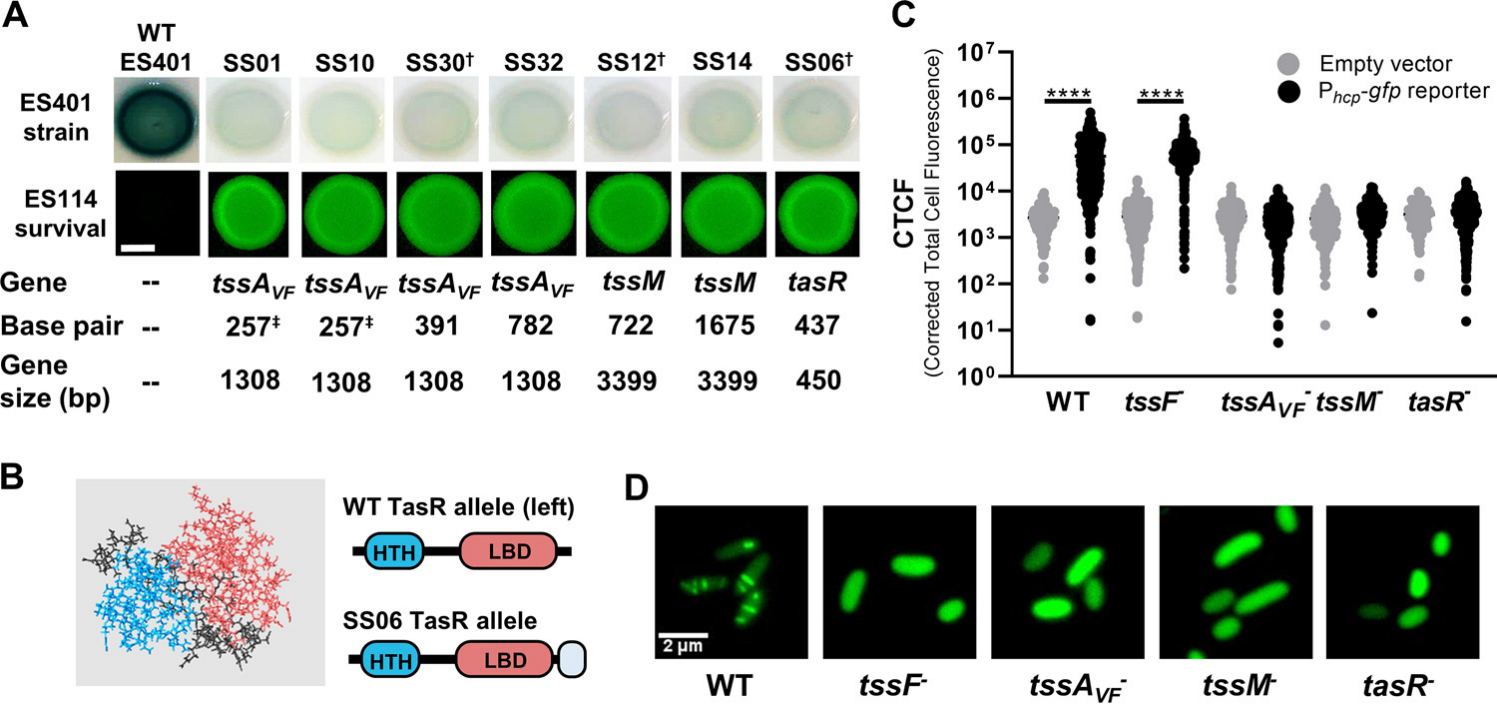
Isolated transposon mutants are unable to activate T6SS transcriptional reporters or build sheaths. (A) Qualitative reporter activity for P*_hcp_-lacZ* in wild-type ES401 and transposon mutants. “Gene” and “Base pair” indicate the location of transposon insertion for each mutant based on the ES401 reference genome (52). Daggers indicate representative mutants from *tssA_VF_*, *tssM*, and *tasR* that were selected for further characterization. Shown are fluorescence microscopy images of ES114 at 24 h following coincubation with each mutant. The scale bar is 2 mm. Images were taken after 24 h on LBS–X-Gal (reporter) or LBS (fluorescence). Assays were performed at least three times (*n* = 4), and a representative experiment is shown. (B) Predicted structure of TasR using Quark (80, 81). Shown are the N-terminal helix-turn-helix domain (blue), C-terminal ligand binding domain (pink), and C-terminal extension in the SS06 mutant (CLLYTSAAALGLAVVLQGP [gray]). (C) Quantitative single-cell reporter activity for P*_hcp_-gfp* in wild-type ES401, a structural *tssF* mutant, and representative regulatory mutants. Cells carrying plasmid pAS2028 were imaged immediately following a 5-h incubation on LBS agar, and the corrected total cell fluorescence (CTCF) was calculated for single cells. The assay was performed two times (*n* > 1,000 cells/treatment), and all data are shown. Circles indicate CTCF measurements from single cells. *, *P* < 0.0001 (one-way analysis of variance [ANOVA] followed by a Tukey’s multiple-comparison test comparing the empty vector to the P*_hcp_*-*gfp* reporter in each strain). (D) Representative green fluorescence images of the ES401 wild-type, a structural *tssF* mutant, and representative regulatory mutant cells carrying the TssB_2-GFP expression vector (pSNS119) after incubation on LBS agar for 5 h. LBS medium was supplemented with 0.5 mM IPTG (isopropyl-β-d-thiogalactopyranoside) to induce expression of TssB_2_GFP.

Strain ES401 carrying the P*_hcp_*-*lacZ* reporter plasmid was mutagenized using the mini-Tn5-*ermR* transposon delivery vector described by Lyell et al. (49) and plated onto Luria-Bertani with added salt (LBS) agar supplemented with X-Gal and the antibiotic erythromycin (Erm). Approximately 20,000 mutants were screened for colony color: seven colonies that appeared white were each given an SS strain name (e.g., SS01, SS10, etc) and selected for further characterization (Fig. 2A). To confirm that the change in phenotype was due to the transposon insertion and not a random mutation elsewhere in the genome, we used natural transformation to reconstruct each of these mutations by moving the Erm-resistant (Erm^r^) transposon insertion from each isolated mutant into a fresh ES401 background and repeated the P*_hcp_*-*lacZ* reporter assay (50). After 24 h of incubation on LBS–X-Gal plates, the ES401 wild-type strain containing P*_hcp_*-*lacZ* was blue; however, the remade ES401 mutant strains harboring the same reporter plasmid appeared white (see Fig. S1 in the supplemental material), indicating that P*_hcp_* was no longer activated in these strains.

Because previous work by Speare et al. (21) revealed a correlation between P*_hcp_*-*lacZ* activity and T6SS2 function, we asked whether these mutants could no longer kill. Coincubation assays were performed as described previously by mixing a fluorescently tagged V. fischeri ES114 target strain with either wild-type or mutant ES401 strains at a 1:1 starting ratio and spotting onto LBS agar plates (21). Fluorescence microscopy was used to qualitatively observe killing by screening for the presence of fluorescently tagged ES114 after a 24-h coincubation period on agar plates. If ES114 is eliminated during the coincubation period, no fluorescence is detectable. After a 24-h coincubation, ES114 was undetectable using fluorescence microscopy when mixed with wild-type ES401, indicating that ES114 was eliminated (Fig. 2A). However, when ES114 was incubated with each of the seven ES401 mutants, fluorescent ES114 was clearly visible in the mixed colony (Fig. 2A). Taken together, these results suggest that mutants that have lost the ability to activate the *hcp* promoter reporter are no longer able to kill a competitor strain.

### Transposon insertions map exclusively to the T6SS2 genomic island.

To identify the insertion sites, genomic DNA (gDNA) from each mutant was used in arbitrarily primed PCR (AP-PCR) or to clone out the transposon, followed by sequencing with transposon-specific primers (51). Sequences were then mapped to the ES401 genome (52). Consistent with our hypothesis that strain-specific reporter activation is due to one or more genes within the genomic island, sequencing revealed that all seven mutants contained a transposon insertion in one of three consecutive genes located on the T6SS2-encoding genomic island. Disruptions were mapped to a gene encoding an ImpA_N domain protein, *tssM* (*icmF*), and a gene encoding a predicted transcriptional regulator, which we term *tasR*, for type VI-associated regulator. Four of these mutants contained insertions in the gene encoding the ImpA_N domain protein (VFES401_15760), while the other transposon insertions mapped to the two genes immediately downstream: *tssM* (VFES401_15765) and *tasR* (VFES401_15770) (Fig. 1A and 2A). Moreover, the presence of multiple transposon insertions at different locations within the same genes suggests this transposon screen successfully saturated the genome.

Our transposon hits included two predicted T6SS structural proteins (ImpA_N domain protein and TssM) and a predicted transcriptional regulator (TasR). ImpA_N domain proteins, which are often referred to as TssA-like proteins, have diverse functions in T6SS assembly and function that appear to be driven by their diversified C-terminal domains (53). For example, the genome of enteroaggregative Escherichia coli (EAEC) encodes two ImpA_N domain proteins: TssA_EC_ and TagA_EC_ (see Fig. S2 in the supplemental material). In EAEC, TssA is essential for T6SS biogenesis, where it associates with the transmembrane structure (TssJLM) to recruit and stabilize the baseplate components (TssEFJK and VgrG) and then coordinates the assembly of the inner tube and outer sheath (Hcp and TssBC) while remaining localized to the distal end of the T6SS sheath during polymerization (54). TagA_EC_, which contains a transmembrane domain and VasJ domain at the C terminus, is tightly associated with the inner membrane to capture the TssA-capped distal end of the sheath, maintaining it in an extended position until fired (55). When we compared the domains and amino acid identities of our V. fischeri TssA-like protein (TssA_VF_) to those of other well-characterized proteins, we found that TssA_VF_ shares less than 15% amino acid identity and it contains a distinct C-terminal domain (Fig. S2), suggesting that the role of this TssA-like protein in V. fischeri may be different from those of previously characterized proteins.

TssM is an essential structural component of the T6SS that is localized to the inner membrane and has been well characterized in other systems (2, 56). Although TssM is not predicted to have a regulatory role, previous studies have shown it has nucleoside triphosphatase (NTPase) activity *in vitro* (56), yet the biological relevance of this enzymatic activity is unknown. When we searched for predicted NTPase domains in TssM protein sequences from V. fischeri ES401 and other vibrios, we found Walker A and B motifs, as well as a GTP-specific binding motif in the ES401 sequence and that of closely related vibrios (see Fig. S3 in the supplemental material).

A Pfam protein sequence analysis (57) indicates TasR is a predicted Lrp/AsnC type regulator with an N-terminal helix-turn-helix domain that is predicted to bind DNA, as well as a predicted C-terminal regulatory ligand-binding domain (Fig. 2B). Lrp/AsnC-type regulators often form dimers or multimers through interactions at the ligand-binding domains, and the conformation of the multimers can change in response to binding small molecules, such as amino acids, which can alter downstream gene regulation (58). Furthermore, previous proteomics experiments indicate that the V. fischeri TasR protein is more abundant in cells grown in hydrogel, compared to low-viscosity liquid, suggesting it is regulated by environmental viscosity (44). A TasR homolog (VP1407) in Vibrio parahaemolyticus is similarly located on a predicted strain-specific genomic island (59) and was previously shown to be necessary for T6SS-dependent killing (60), indicating the importance of this regulator in other systems. Interestingly, the transposon in the *tasR* mutant inserted at the end of the gene, which resulted in a predicted change in the C-terminal sequence, including a 19-amino-acid extension. Given that the C terminus is a predicted ligand-binding domain, we reasoned that the disruption and additional amino acids at the C terminus may inhibit the regulatory function of TasR (Fig. 2B). Further characterizations of how disruptions in these genes impact T6SS expression and function were performed using one representative mutant for each gene: SS30 for *tssA_VF_*, SS12 for *tssM*, and SS06 for *tasR* (Fig. 2A, daggers).

### Transposon mutants are unable to activate a P*_hcp_*-*gfp* reporter or form GFP-tagged sheaths.

Although our initial characterization of the T6SS reporter and function indicates these mutants are impaired at a population level, we set out to investigate these changes at the single-cell level by using green fluorescent protein (GFP)-based reporters of *hcp_2* promoter activity and sheath assembly. We moved either a promoterless *gfp* plasmid (empty vector) or a P*_hcp_*-*gfp* reporter plasmid into wild-type ES401, a structural mutant with a disruption in the *tssF_2* (*vasA_2*) gene (21), which encodes an essential baseplate component (61, 62), and each of our three representative transposon mutants. Following a 5-h incubation on LBS agar to allow for promoter activation, single cells from each treatment were imaged using a fluorescence microscope. Although the wild type and *tssF* structural mutant exhibited significantly higher fluorescence for the reporter compared to the empty vector (Fig. 2C), the *tssA_VF_*, *tssM*, and *tasR* mutants did not exhibit increased reporter activity compared to the empty vector, with reporter activity reduced by ∼94% compared to that of the wild type (Fig. 2C). Taken together, these data suggest that mutants with transposon insertions in the structural genes (*tssA_VF_* and *tssM*) and the regulator (*tasR*) are not able to activate the promoter reporter in any cells within the population.

Given that the *hcp* promoter is not active in these mutants, we hypothesized that these mutants are also unable to build T6SS sheaths. To directly test this hypothesis, we moved a *tssB_2-gfp* expression vector into each of these strains and imaged cells using fluorescence microscopy as previously described (21). The T6SS sheath is composed of TssB (VipA) and TssC (VipB) subunits, and we previously showed that T6SS2 sheaths can be observed in V. fischeri cells expressing TssB-GFP in a *tssF*-dependent manner (21). Although the wild-type control made visible sheaths, the *tssF* baseplate mutant control, as well as all three transposon mutants, was unable to build T6SS2 sheaths, and TssB_2-GFP was dispersed within cells (Fig. 2D).

Based on our initial transposon hits and mutant characterization, we considered two alternative hypotheses to explain why transposon insertions in *tssA_VF_*, *tssM*, and *tasR* prevent *hcp* promoter reporter activation, sheath assembly, and target killing. The first hypothesis is that these three genes are cotranscribed, and only the downstream transcriptional regulator, TasR, is required for P*_hcp_* activation. In this scenario, the transposon insertions in upstream *tssA_VF_* and *tssM* genes merely have a polar effect that impairs *tasR* expression or function. Alternatively, in addition to *tasR*, one or both of the structural proteins may be required for promoter activation.

### Disruptions in *tssA_VF_* and *tssM* do not prevent *tasR* expression.

**FIG 3:**
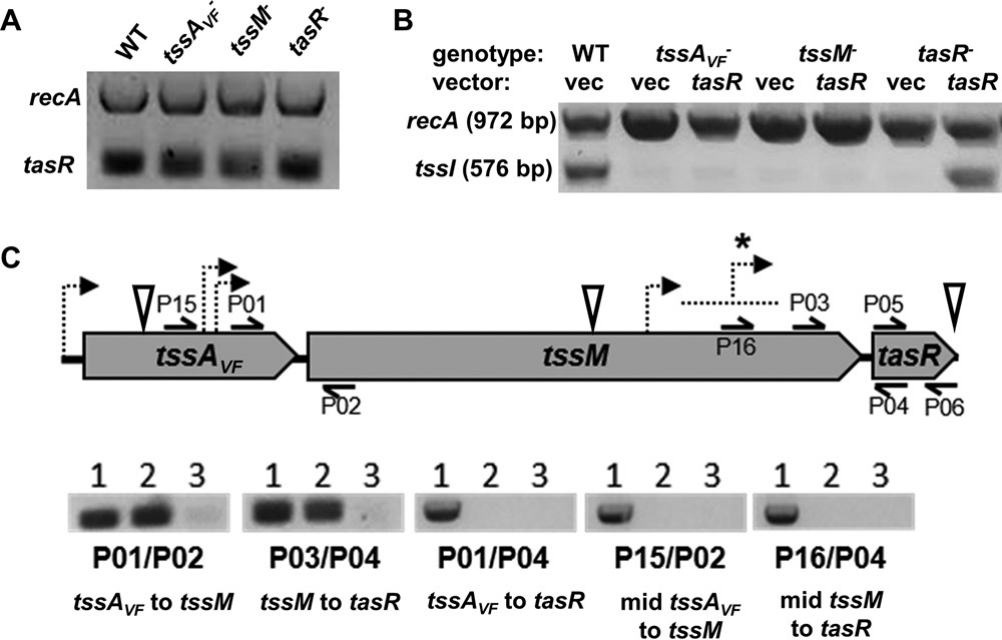
Reverse transcription-PCR indicates that *tssA_VF_*, *tssM*, and *tasR* are not cotranscribed, and transposon insertions in upstream genes do not prevent *tasR* transcription. (A) Gel image depicting transcription of *tasR* and the housekeeping gene *recA* in wild-type ES401 and representative *tssA_VF_*, *tssM*, and *tasR* mutants based on RT-PCR results. RNA was extracted from wild-type or mutant strains following a 5-h incubation on LBS agar plates, and extractions and subsequent transcriptional analysis were performed twice. (B) Gel image depicting transcription of *tssI* and *recA* in wild-type ES401 and representative *tssA_VF_*, *tssM*, and *tasR* mutants carrying either the empty vector (vec) or a *tasR* expression vector. The sequences for each primer (P20/P21) can be found in Table S5. Transcriptional analysis of *recA* was included as a positive control. RNA was extracted following a 5-h incubation on LBS agar supplemented with 0.1 mM IPTG, and extractions and subsequent transcriptional analysis were performed twice. (C) Graphic representation of *tssA_VF_*, *icmF*, and *tasR* genes and the primers used for RT-PCR transcriptional analysis in wild-type ES401. The sequences for each primer can be found in Table S5. Dashed arrows in panel C indicate predicted promoters using the Neural Network Promoter Prediction program set to prokaryotic organisms with a minimum score of 0.9 (64). An asterisk indicates the area where nine predicted promoters were identified. Inverted white triangles indicate transposon insertion sites. Lane assignments in each set: lane 1, wild-type ES401 chromosomal DNA as the template; lane 2, wild-type ES401 cDNA as the template; lane 3, wild-type ES401 cDNA as the template, with no reverse transcriptase. RNA was extracted following a 12-h incubation on LBS agar, and extractions and subsequent transcriptional analysis were performed twice.

To begin testing our first hypothesis, we sought to determine whether the transposon insertions affected our ability to detect *tasR* transcripts by using a reverse transcription-PCR (RT-PCR) approach. RNA was isolated from wild-type and mutant cells, and a one-step RT-PCR was performed using multiplexed primers specific to *recA* and *tasR* sequences. We found that *tasR* transcripts were detectable in all three mutants (Fig. 3A), suggesting that insertions in *tssA_VF_*, *tssM*, and *tasR* (which has the transposon inserted at the end of the gene) do not prevent expression of *tasR*.

### Overexpression of *tasR* is not sufficient to restore T6SS2 gene expression in structural mutants.

To further explore whether *tssA_VF_* and *tssM* disruptions impact T6SS regulation due to an effect on *tasR* expression, we asked whether overexpression of *tasR* in *trans* could restore transcription of T6SS2 genes in our mutants. Due to plasmid incompatibility issues between our reporter and expression vectors, we directly assayed the relative abundance of T6SS2 gene transcripts in strains containing the empty vector or the *tasR* expression vector. Because V. fischeri carries two identical copies of *hcp* that could not be differentiated by RT-PCR, we could not use *hcp* as the transcript to target. However, *tssI* (*vgrG*) is located just downstream of *hcp* on the T6SS2 genomic island, and the two genes have been reported to be cotranscribed in other systems (63). To test whether *hcp* and *tssI* are cotranscribed in V. fischeri, we designed primers that amplify across the *hcp*-*tssI* junction and used either wild-type ES401 genomic DNA (gDNA), RNA, or no reverse transcriptase (RT) as the template in a one-step RT-PCR. We obtained PCR products for the gDNA and RNA template but not for the no-RT control (see Fig. S4A in the supplemental material). This result indicates that *hcp* and *tssI* are cotranscribed in V. fischeri and that *tssI* can be used to assay P*_hcp_* transcriptional activity in our transposon mutants.

Next, we used an RT-PCR approach to assay the relative abundance of *tssI* transcripts in each of our mutants without and with overexpression of *tasR*. If overexpression of *tasR* in the *tssA_VF_* and *tssM* mutants restores *tssI* transcription to wild-type-like levels, this result would support our first hypothesis that disruptions in *tssA_VF_* or *tssM* somehow impact TasR’s ability to activate expression of *hcp*, *tssI*, and downstream structural genes for T6SS assembly and function. However, if *tssI* transcription in these mutants is not restored by *tasR* overexpression, this result would support a possible regulatory role for TssA_VF_ and/or TssM.

Wild-type ES401 and *tssA_VF_*, *tssM*, or *tasR* mutants carrying either an IPTG (isopropyl-β-d-thiogalactopyranoside)-inducible *tasR* expression vector or the empty vector were incubated on LBS agar supplemented with 0.5 mM IPTG for 5 h prior to RNA isolation. Next, we used a one-step RT-PCR kit with multiplexed primers amplifying *recA* and *tssI* to assay T6SS2 transcriptional activity in our mutants without and with expression of *tasR*. Although we obtained PCR products for *recA* in all treatments, we obtained PCR products for *tssI* only in wild-type ES401 and the complemented *tasR* mutant (Fig. 3B). Taken together, these results indicate that overexpression of *tasR* is not sufficient to restore *tssI* expression in *tssA_VF_* or *tssM* mutants.

### Reverse transcription-PCR indicates *tssA_VF_*, *tssM*, and *tasR* are not cotranscribed.

Although the above results are not consistent with possible polar effects of upstream insertions on *tasR*, we next wanted to directly test whether *tssA_VF_*, *tssM*, and *tasR* are possibly cotranscribed. First, we used an RT-PCR approach to experimentally determine the transcriptional units for *tssA_VF_*, *tssM*, and *tasR*. Wild-type ES401 was grown overnight on LBS agar, and total RNA was isolated and converted to cDNA using reverse transcriptase. Standard PCRs performed with primers spanning junctions between *tssA_VF_* and *tssM*, as well as between *tssM* and *tasR*, yielded a PCR product using both gDNA and cDNA template, initially suggesting that these genes may be cotranscribed (Fig. 3C). However, when primers were used to amplify all three genes, PCR product was only observed using gDNA template (Fig. 3C), suggesting that these three genes may not actually be cotranscribed. To resolve this apparent discrepancy, we designed a second set of primers spanning *tssA_VF_* and *tssM*, as well as *tssM* and *tasR*, this time with forward primers that anneal further into the upstream gene sequence (Fig. 3C). When RT-PCR was repeated with this second set of primers, we observed PCR products for only the gDNA template (Fig. 3A).

If these three genes are transcribed on independent transcripts, then we would expect to find predicted promoter sequences for *tssM* and *tasR* in their respective upstream genes. We used the Neural Network Promoter Prediction (NNPP) program (64, 65) to identify high-scoring promoter regions for each gene. Interestingly, we only found two predicted promoter sequences for *tssM* that were located between the two RT-PCR forward primer sites (Fig. 3C). Moreover, the NNPP results identified 10 high-scoring predicted promoters for *tasR* that all mapped to the sequence located between our two forward RT-PCR primer sites (Fig. 3C). Taken together, these results suggest that *tssA_VF_*, *tssM*, and *tasR* are not cotranscribed and that transposon insertions in upstream genes do not prevent *tasR* transcription. (Fig. 3A). Given these findings, we next chose to further explore our second hypothesis—that TasR-dependent activation of the T6SS promoter reporter may require TssA_VF_ and/or TssM.

### Coexpression of all three genes restores killing ability to transposon mutants.

**FIG 4:**
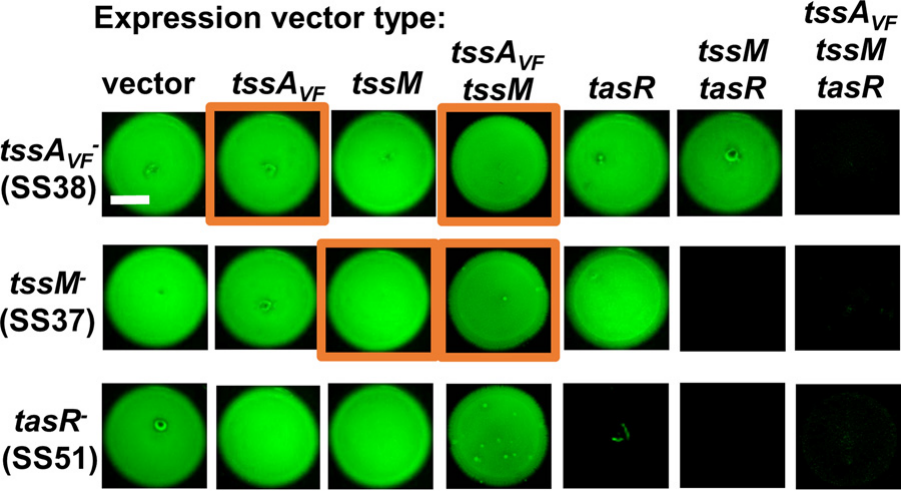
TssA_VF_, TssM, and TasR are all required to restore killing activity in mutants. (A) Fluorescence microscopy images of ES114 at 24 h following coincubation with transposon mutants carrying the empty vector or expression vectors for *tssA_VF_*, *tssM*, *tasR*, *tssA_VF_-tssM*, *tssM-tasR*, or *tssA_VF_-tssM-tasR.* All images were taken after a 24 h incubation on LBS agar supplemented with 0.1 mM IPTG. The scale bar is 2 mm. Assays were performed at least three times (*n* = 3) and a representative experiment is shown.

If our second hypothesis is correct, and both *tssA_VF_* and *tssM* are required for T6SS expression, then we expect in *trans* complementation of the disrupted genes will restore killing function. We generated a series of IPTG-inducible expression vectors for each gene, as well as coexpression vectors of two or all three genes together. The empty vector and expression vectors were each moved into the *tssA_VF_*, *tssM* and *tasR* transposon mutants. Next, we performed coincubation assays between these ES401-derived strains and the fluorescently tagged ES114 target strain, as described above, using plates supplemented with 0.1 mM IPTG to induce gene expression. After 24 h, each coincubation spot was imaged for the presence of the tagged ES114 target. As expected, when the ES401-derived transposon mutants harbored the empty expression vector, ES114 growth was not inhibited (Fig. 4). Surprisingly, single-gene, in *trans* complementation only restored killing ability in the *tasR* mutant, suggesting that expression of the T6SS structural genes alone is not sufficient to restore killing ability for those transposon mutants (Fig. 4). We next tested the ability of our coexpression vectors to restore the ability of these mutants to kill ES114. Coexpression of *tssM* and *tasR* in *trans* restored killing in both *tssM* and *tasR* mutants but not the *tssA_VF_* mutant, while coexpression of all three genes in *trans* restored killing ability in all three transposon mutants (Fig. 4). Taken together, these results suggest that single-gene expression is not sufficient to restore killing in the *tssA_VF_* and *tssM* mutants, and coexpression is required.

### Overexpression of TssA_VF_ and/or TssM prevents killing ability in the wild type.

**FIG 5:**
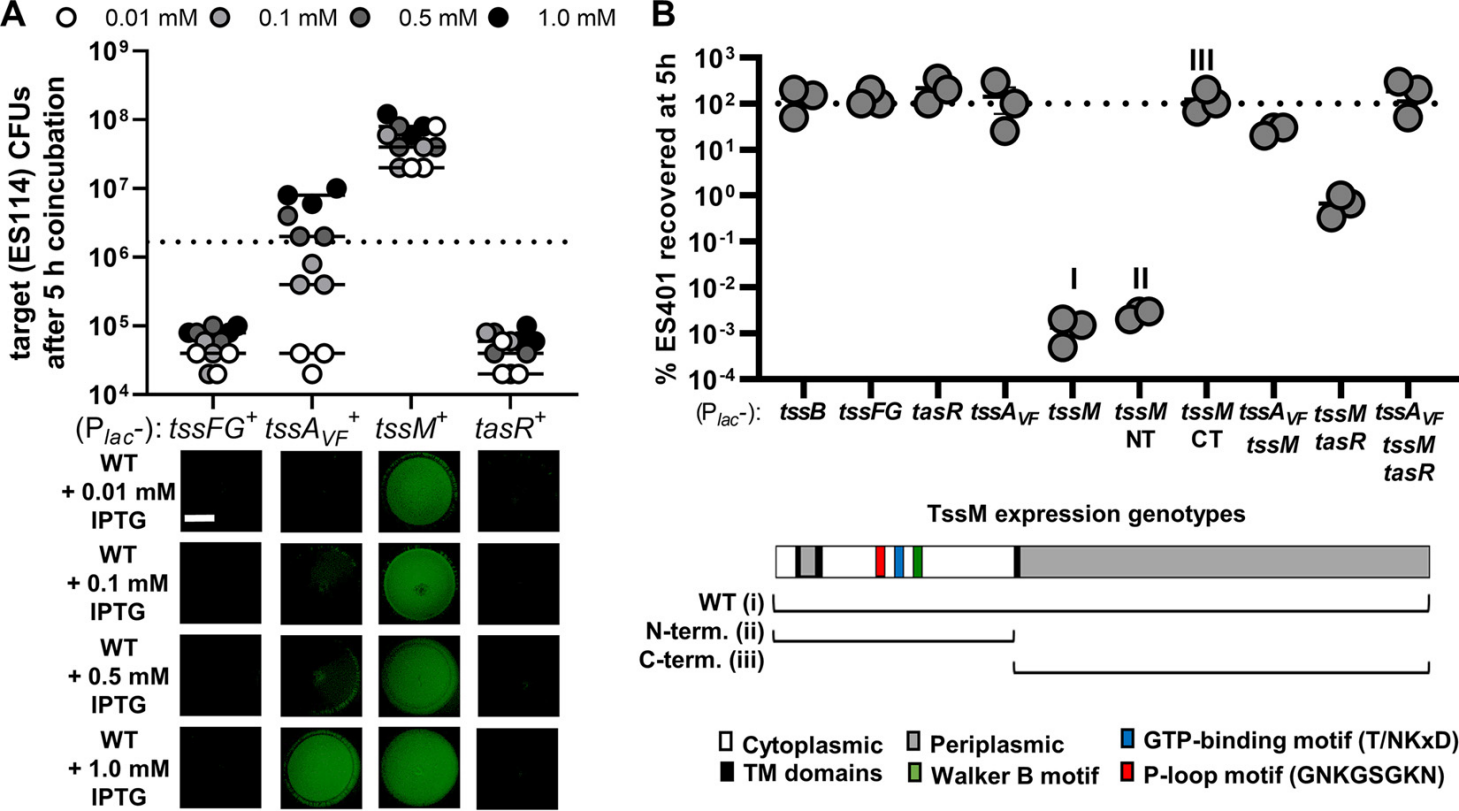
Overexpression of TssA_VF_ or TssM prevents target killing. (A) Total CFU counts of the target strain (ES114) at 5 h and fluorescence microscopy images of ES114 at 24 h following coincubation with wild-type ES401 carrying expression vectors for *tssFG*, *tssA_VF_*, *tssM*, or *tasR* on LBS agar supplemented with either 0.01, 0.1, 0.5, or 1.0 mM IPTG as indicated. The dashed line indicates the average ES114 CFU at 0 h. (B) Percentage of wild-type ES401 CFU recovered after 5 h following an incubation on LBS agar supplemented with kanamycin and 1.0 mM IPTG to induce expression of the indicated genes. The dashed line indicates 100% recovery at 5 h, where >100% indicates net cell growth and <100% indicates net cell death. All assays were performed at least three times (*n* = 3), and a representative experiment is shown. All scale bars are 2 mm.

To help interpret the coincubation assay results where expression of *tssA_VF_* and/or *tssM* in *trans* did not restore killing in the respective mutants, we examined the effect of overexpression of these genes in the wild type. We reasoned that expression of one or more of these genes could inhibit killing ability, and therefore functional complementation of killing would not be possible in these cases. We moved expression vectors for *tssFG* (baseplate components), *tssA_VF_*, *tssM*, or *tasR* into wild-type ES401 and repeated the coincubation assays with fluorescently tagged ES114 target on plates supplemented with different concentrations of IPTG to induce various levels of gene expression. Wild-type cells overexpressing *tssFG* or *tasR* were still able to prevent the growth of tagged ES114 at all concentrations of IPTG (Fig. 5A), indicating overexpression of these proteins does not inhibit T6SS function in the wild type. However, we observed a dose-dependent effect of *tssA_VF_* expression on the ability of the wild type to inhibit ES114 growth, with no ES114 growth inhibition at 1 mM IPTG. For wild-type cells expressing *tssM*, even the smallest amount of IPTG induction (0.01 mM) resulted in growth of ES114 (Fig. 5A). Taken together, these findings indicate that overexpression of some T6SS components, specifically TssA_VF_ and TssM, prevents wild-type cells from killing ES114 target cells, providing some insight into why a single-gene expression vector may not functionally complement the *tssA_VF_* and *tssM* mutants.

We reasoned there were several possibilities for why overexpression of certain T6SS components may prevent the wild-type cells from killing a target. First, protein overexpression could slow the growth of the wild type, making it less efficient at killing a target that can quickly outgrow it. Second, it could impact the expression of T6SS genes. Third, a stoichiometric imbalance of subunits during biogenesis of the T6SS complex could impair function, as reported previously where overexpression of the Vibrio cholerae TagA protein impaired sheath assembly and prevented T6SS-dependent killing of target cells (53). To test the first two predictions, we first quantified the change in cell abundance after 5 h by calculating the percentage of recovery for wild-type cells overexpressing different T6SS components. We found that, like many T6SS proteins, overexpression of *tssA_VF_* did not impact cell growth (Fig. 5B). However, overexpression of *tssM* alone, particularly the N terminus portion that encodes the predicted GTPase domains, caused the cells to die. Coexpression of *tssM* with *tssA_VF_* or *tasR* partially restored survivability, with coexpression of all three genes fully restoring survivability (Fig. 5B). These results suggest that overexpression of *tssA_VF_* does not impact cell growth, but overexpression of the N terminus of *tssM* is lethal to cells, except when coexpressed with *tssA_VF_* and *tasR*. Finally, to determine whether overexpression of *tssA_VF_* turns off T6SS gene expression, we again used our multiplexed RT-PCR assay and found that *tssI* was still expressed at wild-type-like levels when *tssA_VF_* was overexpressed in wild-type cells (Fig. S4B). Taken together, these data suggest that overexpression of *tssA_VF_* or *tssM* alone or together impairs T6SS function, unless these genes are coexpressed with *tasR*, thus explaining why in *trans* expression of *tssA_VF_* or *tssM* alone or together does not allow for functional complementation in the mutants (Fig. 4, orange squares). Furthermore, because the negative effects of *tssA_VF_* and/or *tssM* overexpression can be prevented when these proteins are coexpressed with the regulator *tasR*, these findings suggest that TasR-dependent activation of T6SS activity requires a functional TssA_VF_ and TssM.

### TssA_VF_, TssM, and TasR are all required to activate expression of T6SS structural proteins in hydrogel.

Our data indicate that TssA, TssM, and TasR are all required for activation of expression of key T6SS structural components and sheath assembly. We were therefore interested in identifying the broader regulons that these proteins control in the cellular system. To identify these regulons, we used a proteomics approach on wild-type and mutant cultures grown in liquid hydrogel. Previously, we used proteomics to show that the V. fischeri ES401 T6SS2 proteins are highly expressed and functionally active in a hydrogel medium that mimics the high-viscosity host environment (44). To determine whether our regulatory genes are also active in hydrogel, we performed coincubation assays between wild-type ES401, a *tssA_VF_*, *tssM*, or *tasR* mutant, and the ES114 target strain. Strains were coincubated in a hydrogel consisting of liquid medium supplemented with polyvinylpyrrolidone (PVP) at 5% (wt/vol) for 12 h, and ES114 CFU counts were obtained at 0 h and 12 h by spotting serial dilutions on LBS plates supplemented with antibiotics to select for ES114 target cells. As expected, when ES114 was coincubated with wild-type ES401, we observed a decrease in ES114 CFU after 12 h (see Fig. S5 in the supplemental material). However, ES114 CFU increased from 0 h to 12 h when coincubated with a *tssA_VF_*, *tssM*, or *tasR* transposon mutant, with the final ES114 CFU for mutant coincubations being significantly different from that of the coincubation with the wild type (Fig. S5). These data indicate that hydrogel is an appropriate growth condition to use to probe the regulons of TssA_VF_, TssM, and TasR.

**FIG 6:**
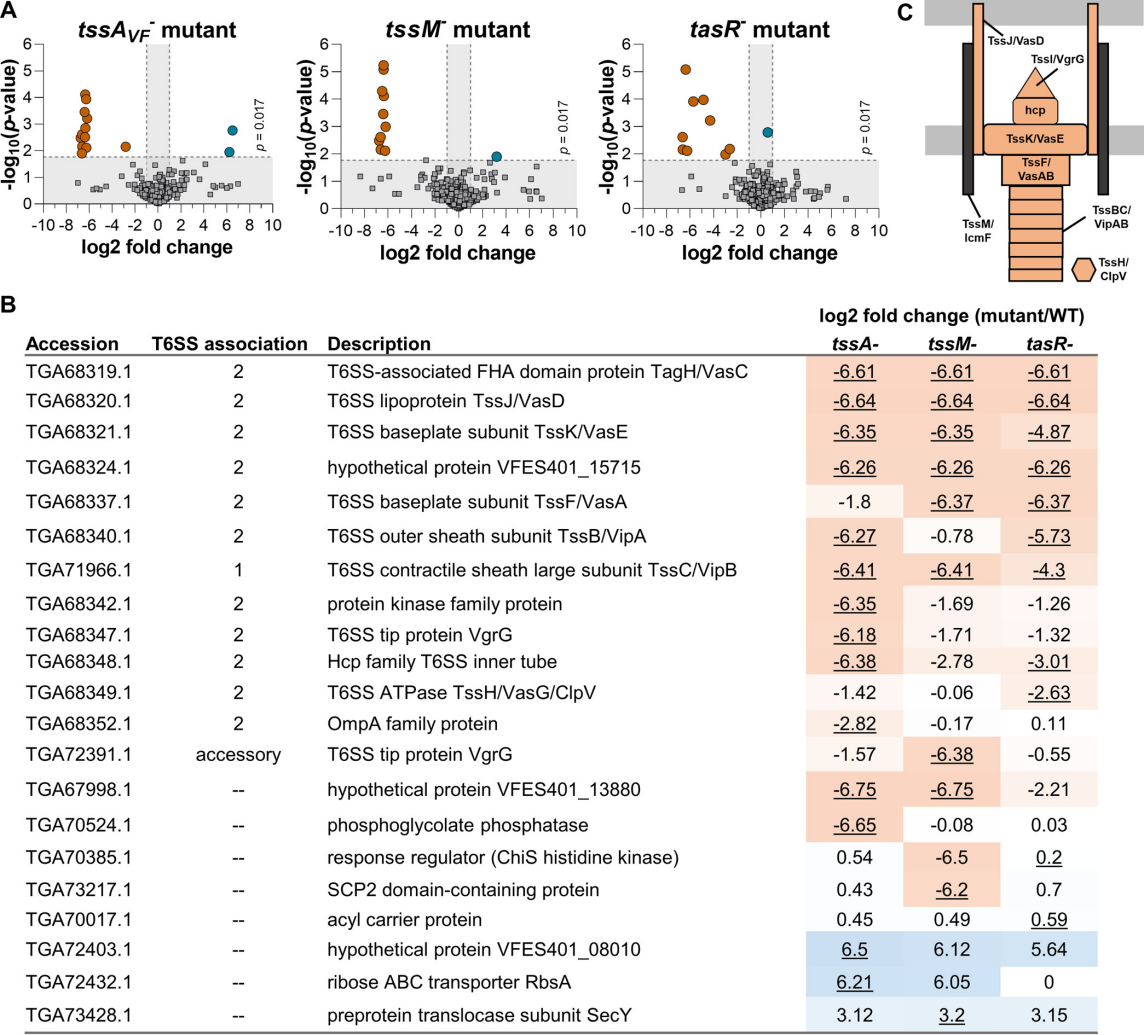
TssA_VF_, TssM, and TasR are required to activate expression of T6SS structural proteins in hydrogel. (A) Volcano plots showing the log_2_ fold difference in protein abundance between the T6SS2 mutant and wild-type ES401. Proteins with a negative log_2_ fold change are more abundant in wild-type ES401 (left; orange), and proteins with a positive log_2_ fold change are more abundant in the mutant (right [blue]). Data points above the dashed line had significant *P* values between treatments (Student's *t* test with the Bonferroni correction, *P* < 0.017), and those outside the vertical dashed lines had a magnitude fold change of >1 log_2_ between treatments. (B) Table describing the log_2_ fold change (mutant versus WT) of all proteins that were significantly differentially expressed between the mutant and wild type in at least one treatment. Underlined log_2_ fold change indicates significantly differently expressed compared to the wild type (Bonferroni corrected α = 0.017). NSAF values of zero were replaced with the limit of detection (0.000762) for calculation of log_2_ fold change values. Heat map colors were assigned expression levels of high (orange) to low (blue) based on log_2_ fold change values. (C) Structural T6SS2 proteins. Orange indicates that the protein was differentially expressed between wild-type ES401 and at least one of the regulatory mutants (Student's *t* test with the Bonferroni correction, *P* < 0.017).

We next used a proteomics approach to quantify differences in protein abundance comparing wild-type ES401 and the *tssA_VF_*, *tssM*, and *tasR* mutants. Each strain was grown in hydrogel for 24 h before cells were harvested and frozen for protein extraction, as described previously (44). We detected 1,394 proteins, or 36% of the proteins encoded in the ES401 genome (52). We then compared the proteome of each mutant to that of wild-type ES401 and constructed volcano plots of the resulting differentially abundant proteins. Proteins that were significantly more abundant in the wild-type strain compared to the mutant are shown as orange circles, and proteins that were significantly more abundant in the mutant are shown as blue circles (Fig. 6A). All other proteins that did not pass our threshold values for being significantly differentially abundant are shown as gray squares. Only 11 to 14 proteins were differentially abundant comparing wild-type and mutant treatments, with the majority of these proteins being T6SS components (Fig. 6B and C; see Tables S1 to S4 in the supplemental material). Together, these data suggest that in addition to their structural roles in T6SS function, TssA_VF_ and TssM, along with the regulator TasR, are required for expression of a relatively small regulon composed primarily of the T6SS components necessary for killing competitor strains.

## DISCUSSION

This study aimed to identify essential regulators of the promoter that activates expression of T6SS2 structural proteins. Our global search for regulators yielded a surprising result: two broadly conserved structural proteins are required for T6SS protein expression. Interestingly, our previous work showed that although the majority of T6SS-associated proteins were significantly more abundant in hydrogel compared to liquid, including the essential regulator TasR, peptides specific to TssA_VF_ and TssM were not differentially abundant under either condition (44), suggesting these proteins are constitutively expressed. Moreover, the V. fischeri TssM protein contains predicted GTPase-related domains that are conserved in other TssM homologs (see Fig. S3 in the supplemental material), and work with Agrobacterium tumefaciens showed TssM has ATPase activity *in vitro* (56), providing further support that these domains have an unknown biological function. Finally, TssA_VF_ contains a predicted GTP binding domain and a unique C-terminal region that is not found in other well-characterized TssA-like proteins (Fig. S2).

The presence of GTP-related domains in proteins that are required for expression of other T6SS structural proteins provides a connection, albeit a speculative one, between sensing of cellular energy levels and expression of this costly competitive mechanism. Although future work will be required to understand how cells may ensure the T6SS is expressed only when cellular energy stores are sufficient to support its assembly and deployment, TssA_VF_ and TssM would appear to be logical targets of future exploration. Indeed, prokaryotic GTPases are known to play roles in diverse cellular processes, including the stringent response (66). When nutrients are low, cells produce the signaling molecule (p)ppGpp, which is derived from GDP and GTP. In E. coli, (p)ppGpp inhibits the GTPase activity of Der, possibly by binding to the GTP binding site, which regulates its activity under stress conditions (67). If the TssM GTPase motifs are functional in the cell, it seems reasonable to hypothesize a similar mechanism for T6SS regulation, where the cell uses a constitutively expressed essential structural protein to sense the intracellular energy levels to prevent expression of the T6SS when nutrients are limiting. Future work that focuses on determining the activity of these predicted GTPase-related domains, as well as the subcellular locations and possible physical interactions among these proteins, will be essential to fully understand the roles of these proteins in T6SS regulation and function.

Our proposed model, which posits that the constitutively expressed TssA_VF_ and TssM proteins play a key role in activating T6SS expression, would be particularly important for activating T6SS expression if the GI is transferred into a new bacterial host. Indeed, the ability of genes on mobile elements to self-regulate is not without precedent. For example, the predicted ancestors of T6SSs, phages, are known to self-activate gene expression as lytic viruses and as prophages that self-activate in response to stimuli such as DNA damage (40). Moreover, recent work in Burkholderia thailandensis demonstrated that another contact-dependent inhibitory system, known as CDI, which is encoded in the *bcp* genes, is located on a new class of transposable elements (68). When the mobile element transfers into a new bacterial host, it brings with it the genes necessary for activation of the CDI system. Here, we present preliminary data that are consistent with a similar mechanism of self-activation for T6SSs encoded on a genomic island. However, future work is needed to understand the molecular mechanism by which TssA_VF_ and TssM promote T6SS expression and to determine how this possible self-activation mechanism is integrated into other species-specific regulatory networks to optimize T6SS expression and enhance competition for a specific niche.

## MATERIALS AND METHODS

### Media and growth conditions.

V. fischeri strains were grown in LBS medium at 24°C, and E. coli strains were grown in either LB medium or brain heart infusion (BHI) (Difco) at 37°C. Antibiotic selection for V. fischeri and E. coli strains was described previously (69). Briefly, for selection in E. coli cultures, chloramphenicol (Cm) and kanamycin were added to LB medium at final concentrations of 20 and 40 μg ml^−1^, respectively, and erythromycin (Erm) was added to BHI medium at a final concentration of 150 μg ml^−1^. For selection in V. fischeri cultures, chloramphenicol, kanamycin, and erythromycin were added to LBS medium at final concentrations of 2, 100, and 5 μg ml^−1^, respectively. Plasmids with the R6Kγ origin of replication were maintained in E. coli strain DH5α λpir (69), and plasmid pEVS104 (70) was maintained in strain CC118 λpir (71). All other plasmids were maintained in E. coli strain DH5α (72).

### Molecular techniques.

PCR was performed on a Mastercycler Pro (Eppendorf) using Phusion high-fidelity DNA polymerase (New England Biolabs), except where noted. PCR products were purified using the DNA Clean and Concentrater-5 kit (Zymo Research, Orange, CA). Plasmids were isolated using the ZR Plasmid Miniprep Classic kit (Zymo Research, Orange, CA). The DNA concentration was measured with a BioSpectrometer Basic (Eppendorf). Sequencing was performed through Eton Biosciences or GENEWIZ and analyzed using A plasmid Editor (ApE).

### Transposon mutagenesis.

Triparental matings were set up between V. fischeri strain ES401 carrying P*_hcp_-lacZ-*reporter pAG01, the mini-Tn5-*ermR* transposon vector pEVS170, and conjugative helper pEVS104 as previously described (49, 70). After a 24-h incubation period on LBS agar at 24°C, mating spots were resuspended in 1 ml LBS medium and spread plated onto LBS agar medium containing Erm and 50 μg ml^−1^ 5-bromo-4-chloro-3-indolyl-β-d-galactoside (X-Gal). A total of ∼20,000 mutant colonies were screened for blue or white coloration.

**(i) Arbitrarily primed PCR.** For the first round of arbitrarily primed PCR (AP-PCR), the transposon-specific primer 170Int2 and a random oligonucleotide, ARB1, were used to amplify DNA extracted from ES401 transposon mutants. Because of the low stringency that results from using random oligonucleotides, a second round of PCR was performed. This reaction used a second transposon-specific primer and random oligonucleotide (170Ext3/ARB2), which further enriched the amplification of the transposon insertion junction (73). To identify the site of transposon integration, the AP-PCR product for ES401 mutants SS01 and SS32 was sequenced using primer 170EXT and mapped using V. fischeri ES401 as a reference genome.

**(ii) Cloning into DH5α λpir.** Transposon insertion sites for ES401 mutants SS06, SS10, SS12, SS14, and SS30 were determined by cloning out the transposon and flanking DNA and then sequencing across the transposon-chromosome junction using primer M13F. Briefly, chromosomal DNA was digested with HhaI restriction enzyme, and fragments were self-ligated with T4 DNA ligase. The resulting circularized transposon and flanking DNA was transformed into E. coli strain DH5α λ*pir* and selected for using the Erm resistance gene contained within the transposon (74).

### Strain and plasmid construction.

The bacterial strains, plasmids, and oligonucleotides used in this study are presented in Table S5 in the supplemental material. The resulting plasmids were mobilized into recipients by triparental mating using CC118 λpir/pEVS104 as a conjugative helper. All primer design was based on the MJ11 genome sequence, unless otherwise noted, and primers were synthesized by Eton Biosciences or Integrated DNA Technologies (IDT).

To reconstruct ES401 transposon mutants in a fresh wild-type background, genomic DNA was isolated from overnight cultures of mutant strains grown in LBS at 24°C. Overnight cultures of wild-type ES401 carrying the pLosTfoX plasmid were grown in Fischeri minimal medium (FMM) supplemented with Cm and *N*-acetylglucosamine (GlcNAc; 10 μM). Natural transformation was performed according to Pollack-Berti et al. (50). Erm^r^ colonies were considered transformants and were referred to as “reconstructed mutants” in subsequent experiments.

To construct an expression vector for the *tasR* mutation, *tasR* was PCR amplified from strain ES401 gDNA using primers ANS1167 and P27. The forward primer includes 21 bp upstream of the start codon to include the native ribosome binding site (RBS). The reverse primer included the native stop codon to prevent a translational fusion to, or expression of, the downstream *gfp* gene on pAKD601. The resulting *tasR* PCR product was cloned into the KpnI- and XbaI-cut sites of plasmid pAKD601, located downstream of an IPTG-inducible promoter, resulting in plasmid pSNS111.

To construct an expression vector for the *tssA_VF_* mutation, *tssA_2* was PCR amplified from strain ES401 gDNA using primers P103 and P104. The forward primer included a 15-bp region encoding an RBS, and the reverse primer included the native stop codon to prevent a translational fusion to, or expression of, the downstream *gfp* gene on pAKD601. The resulting *tssA_2* PCR product was cloned into the KpnI- and XbaI-cut sites of plasmid pAKD601, located downstream of an IPTG-inducible promoter in plasmid, resulting in plasmid pSNS114.

To construct an expression vector for both the *tssM* and *tasR* genes, *tssM_2* and *tasR* were PCR amplified together from strain ES401 gDNA using primers P78 and P71. The forward primer includes 8 bp upstream of the start codon to include the native RBS. The reverse primer includes the native stop codon to prevent a translational fusion to, or expression of, the downstream *gfp* gene on pAKD601. The resulting *tssM_2-tasR* PCR product was cloned downstream of an IPTG-inducible promoter in plasmid pAKD601 (cut with KpnI and NheI) using the standard SLIC cloning technique (75), resulting in plasmid pSNS126.

To construct an expression vector for the *tssM* mutation, the plasmid pSNS126 was PCR amplified using primers SNS95 and SNS96, which were designed to amplify around the plasmid and exclude the *tasR* gene. The resulting pSNS126 PCR product was phosphorylated using T4 polynucleotide kinase (PNK), and a DpnI digest was performed to eliminate any remaining methylated template vector. This product was ligated and transformed into DH5α λpir, resulting in vector pSNS131.

To construct an expression vector for both the *tssA_VF_* and *tssM* mutations, *tssA_2* and *tssM_2* were PCR amplified together from strain ES401 gDNA using primers P117 and P119. The forward primer included a 15-bp region encoding the RBS sequence (AGGAGGAAATTAAGC). The reverse primer included the native stop codon to prevent a translational fusion to, or expression of, the downstream *gfp* gene on pAKD601. The resulting *tssA_2-tssM_2* PCR product was cloned downstream of an IPTG-inducible promoter in plasmid pAKD601 (cut with KpnI and NheI) using the standard SLIC cloning technique (75), resulting in plasmid pSNS142.

To construct an expression vector for the *tssA_VF_*, *tssM*, and *tasR* mutations, *tssA_2*, *tssM_2*, and *tasR* were PCR amplified together from strain ES401 gDNA using primers P103 and P27. The forward primer included a 15-bp region encoding the RBS from plasmid pTM214. The reverse primer included the native stop codon to prevent a translational fusion to, or expression of, the downstream *gfp* gene on pAKD601. The resulting *tssA_2-tssM_2-tasR* PCR product was cloned downstream of an IPTG-inducible promoter in plasmid pAKD601 (cut with KpnI and NheI) using the standard SLIC cloning technique (75), resulting in plasmid pSNS143.

To construct the *gfp*-based hcp_2 promoter reporter, the *hcp_2* promoter sequence was PCR amplified from plasmid pAG01 using primers AS1094 and AS1095. Plasmid pJLS71 (ref PMID 29752265) was PCR amplified using primers AS1092 and AS1093. PCR products were digested with DpnI to remove methylated template, and resulting PCR products were combined with SLIC, as described above, resulting in plasmid pAS2028.

### *lacZ-*based reporter assays.

V. fischeri strains carrying the P*_hcp_-lacZ* reporter were grown overnight on LBS agar plates supplemented with the appropriate antibiotic at 24°C. Cells were scraped from agar surfaces, resuspended in LBS medium, and diluted to an optical density at 600 nm (OD_600_) of 1.0. For each strain, 5 μl of culture was spotted on LBS agar plates containing 1.0 mM X-Gal and incubated at 24°C. After 24 h, representative spots from each strain were imaged using a Pluggable USB2-MICRO-250× digital microscope. Each experiment was repeated three times with four independent cultures of each strain.

### Coincubation assays.

V. fischeri strains containing either pVSV102 (GFP) or pVSV208 (dsRed) were grown overnight on LBS agar plates supplemented with the appropriate antibiotic at 24°C. Cells were scraped from agar surfaces, resuspended in LBS medium, and diluted to an OD_600_ of 1.0. For each coincubation, strains were mixed in a 1:1 ratio, and either 5 μl of the mixture was applied as spots onto either LBS agar plates supplemented with IPTG when needed, or cells were mixed in 5% PVP hydrogel medium as described by Speare et al. (44) and incubated at 24°C. After 5 h, coincubations from LBS agar were resuspended in 1 ml LBS medium. Strains were quantified by plating serial dilutions for T0 (initial) and T-Final (resuspended after coincubation period) onto LBS plates supplemented with antibiotics selective for each strain. Each experiment was repeated four times with three independent cultures of each strain.

### Fluorescence microscopy.

For each coincubation assay, fluorescence microscopy images were taken with a trinocular zoom stereo microscope equipped with a Nightsea fluorescence adapter kit for green and red fluorescence detection. Images were taken using an OMAX 14MP camera with OMAX ToupView camera control software. Color changes were made by adjusting the lookup value to “Green” in FIJI. No brightness or contrast adjustments were made. For single-cell imaging of T6SS transcriptional reporter and TssB_2-GFP sheaths, images were taken with an Olympus BX51 microscope outfitted with a Hamamatsu C8484-03G01 camera and a 100×/1.30 Oil Ph3 lens objective as described previously (21).

### Reverse transcription-PCR.

RNA was extracted from wild-type or mutant V. fischeri strains following incubation for 5 or 12 h at 24°C on LBS agar plates supplemented with the appropriate antibiotic and IPTG when indicated. RNA extractions were performed according to the ZR Fungal/Bacterial RNA Miniprep kit (Zymo Research, Orange, CA). Transcriptional analysis was performed according to the ONETAQ One-Step RT-PCR kit (NEB, Ipswich, MA), and similar concentrations of DNA, RNA, or cDNA were added to PCR mixtures within experiments. All RT-PCR experiments were performed on two separate occasions with RNA derived from separate extractions, each with similar results.

### Sample preparations for proteomics.

We performed sample preparation as described by Speare et al. (44). Briefly, we added 60 μl of SDT lysis buffer (4% [wt/vol] SDS, 100 mM Tris-HCl, 0.1 M dithiothreitol [DTT]) to each cell pellet and then incubated the samples at 95°C for 10 min for cell lysis. Tryptic digests of protein extracts were prepared following the filter-aided sample preparation (FASP) protocol described by Wisniewski et al. (76). In addition to minor modifications as described in Kleiner et al. (77), we loaded the whole lysate on to the filter units used for the FASP procedure. Peptide concentrations were determined with the Pierce Micro BCA (bicinchoninic acid) assay (Thermo Scientific) using an Epoch2 microplate reader (Biotek) following the manufacturer’s instructions.

### LC-MS/MS.

Four hundred nanograms of peptides for each sample was analyzed by one-dimensional liquid chromatography-tandem mass spectrometry (1D LC-MS/MS) as described by Speare et al. (44). Briefly, peptides were loaded with an UltiMate 3000 RSLCnano liquid chromatograph (Thermo Fisher Scientific) in loading solvent A (2% acetonitrile, 0.05% trifluoroacetic acid) onto a 5-mm, 300-μm ID C_18_ Acclaim PepMap100 precolumn (Thermo Fisher Scientific). Peptides were separated on the analytical column (75-cm by 75-μm analytical EASY-Spray column packed with PepMap RSLC C_18_, 2-μm material; Thermo Fisher Scientific) by using a 140-min gradient, and mass spectrometry analyses was performed on a Q Exactive HF hybrid quadrupole Orbitrap (Thermo Fisher Scientific) are described by Speare et al. (44).

### Protein identification and statistical analysis.

A database containing protein sequences from V. fischeri ES401 (SRJG00000000.1) downloaded from NCBI (https://www.ncbi.nlm.nih.gov/Traces/wgs/SRJG01) was used. Sequences of common laboratory contaminants were included by appending the cRAP protein sequence database (http://www.thegpm.org/crap/). The final database contained 3,925 protein sequences. Searches of the MS/MS spectra against this database were performed with the Sequest HT node in Proteome Discoverer version 2.2.0.388 (Thermo Fisher Scientific) as described by Speare et al. (44). Only proteins identified with medium or high confidence were retained, resulting in an overall false-discovery rate of <5%. For protein quantification, normalized spectral abundance factors (NSAFs) (78) were calculated and multiplied by 100 to obtain the relative percentage of protein abundance.

Contingency tables were generated by comparing average protein abundance for the wild type compared to each mutant strain using Student’s *t* test, corrected for multiple comparisons using a Bonferroni correction. Volcano plots were generated for each mutant strain by graphing the negative log_10_ *P* value and log_2_ fold change. NSAF values of 0 were replaced by the limit of detection value of 0.000762 to allow for comparisons of the log_2_ fold change between treatments.

### Data availability.

The mass spectrometry proteomics data and protein sequence database have been deposited in the ProteomeXchange Consortium via the PRIDE (79) partner repository under the data set identifier PXD017722.
