# Supplementary material for: Activation of the Type VI Secretion System in the Squid Symbiont Vibrio fischeri Requires the Transcriptional Regulator TasR and the Structural Proteins TssM and TssA
Source: J Bacteriol. 2021 Oct 12;203(21):e00399-21. doi: 10.1128/JB.00399-21 (PMC8508121; doi:10.1128/JB.00399-21)
Supplement: Supplemental file 5 — Fig. S1 to S5 and Table S5. Download JB.00399-21-s0005.pdf, PDF file, 0.3 MB [file jb.00399-21-s0005.pdf]

Supplemental Figure Legends

Figure S1. Reconstructed transposon mutants are unable to activate T6SS transcriptional reporter. Qualitative reporter activity for P_{hcp}-*lacZ* in wild-type ES401 and each transposon mutant reconstructed in an ES401 background. Scale bar is 2 mm. Images taken after 24 h on LBS-X-Gal. Assays were performed at least three times with four biological replicates and a representative experiment is shown.

Figure S2. The *Vibrio fischeri* ImpA_N domain protein is distinct from TssA and TagA homologs. Domain architecture for *V. fischeri* ES401 $TssA_{VF}$ and ImpA_N domain proteins whose T6SS-related functions have been characterized in other species were identified using Hmmer. The GTP-binding motif (T/NKxD) was located by a manual sequence search and is only found in *V. fischeri* $TssA_{VF}$ and not found in homologs outside of the *V. fischeri* species. Percent identity to the ES401 protein was calculated by aligning the full-length proteins using Clustal Omega.

Figure S3. The *V. fischeri* TssM protein contains predicted GTPase domains. Walker A, Walker B, and GTP-specific binding motifs were identified by searching the ES401 TssM sequence manually. Analogous regions were identified by aligning the full-length sequences of ES401 TssM with closely-related vibrio homologs using Clustal Omega and visualized using Geneious. Black boxes outline motif regions and amino acid letter shading indicates conservation across species (black shading indicates a residue is 100% conserved).

Figure S4. Co-transcriptional analysis of wild-type ES401 T6SS2 genes. (A) Graphic representation of *hcp* and *tssI* genes and the primers used for RT-PCR transcriptional analysis in wild-type ES401. The sequences for each primer can be found in Supplemental Table 5. Dashed arrows indicate predicted transcription based on RT-PCR results. Gel image depicting co-transcription of *hcp* and *tssI* in wild-type ES401 using primers P20 and P22. The lane assignments

are as follows (1) wild-type ES401 chromosomal DNA as template; (2) wild-type ES401 RNA as template; (3) wild-type ES401 RNA as template, no reverse transcriptase. (B) Gel image based on RT-PCR results depicting relative abundance of *recA* and *tssI* transcripts in either wild-type ES401 carrying the empty vector (vec), a $tssA_{VF}$ expression vector, a $tssA_{VF}$-*tssM*-*tasR* expression vector, or the $tssA_{VF}^{-}$ mutant as a negative control. RNA was extracted from each strain following a 5 h incubation on LBS agar plates supplemented with 1.0 mM IPTG, and extractions and subsequent transcriptional analysis was performed twice.

Figure S5. Tn mutants remain non-lethal in high-viscosity media. CFUs of ES114 at T0 (gray) or following a 12 h (green) coincubation with wild-type (WT) or mutant ES401 strains in a high-viscosity (hydrogel) liquid medium. *, $P<0.0001$ by a 2-way ANOVA followed by a Sidak's multiple comparisons test comparing 12 h CFUs from coincubation with each mutant to coincubation with WT ES401. Experiment was performed 3 times and combined data are shown (n = 12).

Supplemental Table S5. Strains, Plasmids, Oligos table.

Strains or plasmids	Relevant characteristics	Source or Ref.
E. coli		
DH5α	F'/*endA1 hsdR17 glnV44 thi-1 recA1 gyrA relA1* Δ(*lacIZYA-argF*)*U169deoR*(f80*dlacI*Δ(*lacZ*)*M15*)	Hanahan, 1983
DH5αλ pir	λ*pir* derivative of DH5α	Dunn et al, 2005
CC118λpir	D(*ara-leu*) *araD* D*lac74 galE galK phoA20 thi-1 rpsE rpsB argE*(Am) *recA* λ*pir*	Herrero et al, 1995
V. fischeri		
ES114	Wildtype *V. fischeri* isolated from *E. scolopes* light organ	Boettcher and Ruby, 1990
PP3	Wildtype *V. fischeri* isolated from Kaneohe Bay water	Lee and Ruby, 1992
ES213	Wildtype *V. fischeri* isolated from *E. scolopes* light organ	Boettcher and Ruby, 1990
MB13B2	Wildtype *V. fischeri* isolated from *E. scolopes* light organ	Wollenberg and Ruby, 2009
H905	Wildtype *V. fischeri* isolated from Kaneohe Bay water	Lee and Ruby, 1992
FQ-A001	Wildtype *V. fischeri* isolated from *E. scolopes* light organ	Sun and Miyashiro, 2016
MJ11	Wildtype *V. fischeri* isolated from *M. japonicus* light organ	Ruby and Nealson, 1976
ES401	Wildtype *V. fischeri* isolated from *E. scolopes* light organ	Fidiopastis et al, 2002
SS01	ES401 with Tn insertion at 257 bp in *tssA* (Em^R)	This study
SS10	ES401 with Tn insertion at 257 bp in *tssA* (Em^R)	This study
SS30	ES401 with Tn insertion at 391 bp in *tssA* (Em^R)	This study
SS32	ES401 with Tn insertion at 782 bp in *tssA* (Em^R)	This study
SS12	ES401 with Tn insertion at 722 bp in *tssM_2* (Em^R)	This study
SS14	ES401 with Tn insertion at 1675 bp in *tssM_2* (Em^R)	This study
SS06	ES401 with Tn insertion at 437 bp in *tasA* (Em^R)	This study
SS35	Reconstructed ES401 with *tssA*::Tn from SS01 (Em^R)	This study
SS33	Reconstructed ES401 with *tssA*::Tn from SS10 (Em^R)	This study
SS39	Reconstructed ES401 with *tssA*::Tn from SS32 (Em^R)	This study
SS34	Reconstructed ES401 with *tssM_2*::Tn from SS14 (Em^R)	This study
SS38	Reconstructed ES401 with *tssA*:: Tn from SS30 (Em^R)	This study
SS37	Reconstructed ES401 with *tssM_2*::Tn from SS12 (Em^R)	This study
SS51	Reconstructed ES401 with *tasR*:: Tn from SS06 (EmR)	This study
ANS2100	ES401 with *tssF_2* disruption (Em^R)	Speare et al, 2020
**Plasmids**		
pAKD701	Promoterless *lacZ*-based reporter plasmid; *lacZ, oriV*$_{R6K\gamma}$*, oriT,* Kn^R	Dunn et al, 2006
pAG01	*lacZ*-based T6SS reporter; P_{hcp}-*lacZ*, *oriV*$_{R6K\gamma}$*, oriT,* Kn^R	Speare et al, 2020
pJLB207	Consensus promoter driving *lacZ*, P_{con}-*lacZ*, *oriV*$_{R6K\gamma}$*, oriT,* Kn^R	Bose et al, 2011
pEVS170	Mini-Tn5 delivery vector; *oriV*$_{R6K\gamma}$*, tnp*, *oriT,* Kn^R, Em^R	Lyell et al, 2008

pLosTfoX	Plasmid for natural transformations; 995 bp of *V. fischeri* ES114 DNA containing the tfoX^{VF} ORF, Cm^R	Pollack-Berti et al, 2010
pEVS104	Conjugative helper, *oriV*$_{R6K\gamma}$, *oriT*, Kn^R	Stabb and Ruby, 2002
pVSV102	*gfp+, oriV*$_{R6K\gamma}$, *oriV*$_{pES213}$, *oriT*, Kn^R	Dunn et al, 2006
pVSV208	*dsRed+, oriV*$_{R6K\gamma}$, *oriV*$_{pES213}$, *oriT*, Cm^R	Dunn et al, 2006
pAS2028	*gfp*-based T6SS reporter; P$_{hcp}$-*gfp, oriV*$_{R6K\gamma}$*, oriT,* Kn^R	This study
pJLS71	Promoterless Cm^R-*gfp*-based reporter plasmid; *mCherry, oriV*$_{R6K\gamma}$*, oriT,* Kn^R	Stoudenmire et al, 2018
pAKD601	*oriV*$_{R6K\gamma}$, *oriT, lacI*^{q} and IPTG-inducible promoter with optional *gfp* fusion, Kn^R	Dunn and Stabb, 2008
pSNS119	*tssB_2-gfp* fusion expression vector; *oriV*$_{R6k\gamma}$, *oriV*$_{pES213}$*, oriT, Kn*^{R}	Speare *et al.,* 2018
pSNS114	*tssA* expression vector; *oriV*$_{R6K\gamma}$, *oriT, lacI*^{q}, Kn^R	This study
pSNS131	*tssM* expression vector; *oriV*$_{R6K\gamma}$, *oriT, lacI*^{q}, Kn^R	This study
pSNS143	*tssA-tssM* expression vector; *oriV*$_{R6K\gamma}$, *oriT, lacI*^{q}, Kn^R	This study
pSNS111	*tasR* expression vector; *oriV*$_{R6K\gamma}$, *oriT, lacI*^{q}, Kn^R	This study
pSNS126	*tssM-tasR* expression vector; *oriV*$_{R6K\gamma}$, *oriT, lacI*^{q}, Kn^R	This study
pSNS142	*tssA-tssM-tasR* expression vector; *oriV*$_{R6K\gamma}$, *oriT, lacI*^{q}, Kn^R	This study

Oligonucleotides[a]

ARB1	GGCCACGCGTCGACTAGTACNNNNNNNNNNGATAT	Brennan et al, 2013
170Int2	GCAGTTCAACCTGTTGATAGTACG	Brennan et al, 2013
ARB2	GGCCACGCGTCGACTAGTAC	Brennan et al, 2013
170Ext3	CAAAGCAATTTTGAGTGACACAGG	Brennan et al, 2013
M13F	TGTAAAACGACGGCCAGT	Eton Bioscience
170EXT	GCACTGAGAAGCCCTTAGAGCC	Miyashiro et al, 2014
AS1109	CGGGCTTATCGACTGCACGG	This study
AS1110	CCAACATAGTAAGCCAGTAATCG	This study
AS1092	GGATAATAGCATGCCAACTCCAGGATATATCCCAATGGCATCG	This study
AS1093	CTCTGGCTAATGATTGAAACTAACTAACGGTGGTATATCCAGTG	This study
AS1094	CACTGGATATACCACCGTTAGTTAGTTTCAATCATTAGCCAGAG	This study
AS1095	CGATGCCATTGGGATATATCCTGGAGTTGGCATGCTATTATCC	This study
P01	CAGTTACAGTAATTCACTCTTCCC	This study
P02	AACAAGATACACTGGTTTCTTTCC	This study
P03	ACTTTATCTAAAAATGGTCAATGGGC	This study
P04	ATTCGCAATAGACTCTCTACCG	This study
P05	GATATACAGCGCAAATCCGC	This study
P06	TTTTATGCCCGATAACGATTCG	This study

P15	CACTATGGGAACAAAAGCCC	This study
P16	CACAGTTTTCCTCAACGGC	This study
P20	ATGCCAACTCCAGCATATATG	This study
P21	TTGGTCAAGGTGTTGGAAC	This study
P22	GTCCCGAACTTTCCATTTTTTG	This study
P103	GTGGATCCGAGCTCGGTACCAGGAGGAAATTAAGCTATATGGT TTATGTGTCACGTTGTC	This study
P104	AGTTCTTCTCCTTTGCTAGCTTATGCAGCCTTTCTTGTTAACC	This study
P78	GTGGATCCGAGCTCGGTACCAGGAGGAAATAATTATGATTAAA AATACTTTGGCTAGAACACC	This study
P71	AGTTCTTCTCCTTTGCTAGCTTATTTTTTGTTTATTACTTCTGGTA GCTTAAA	This study
P117	GTGGATCCGAGCTCGGTACCAGGAGGAAATTAAGCATGCTAGG TTTAAAGTTTAATATGAATCT	This study
P119	AGTTCTTCTCCTTTGCTAGCTTATTTTTTGTTTATTACTTCTGGTA GCTT	This study
AS1167	ATGGTACCCCTATAAAGGCAGGGAACAAC (KpnI)	This study
P27	ATTCTAGATTATCGACACACTGGCTGG	This study
P77	AGTTCTTCTCCTTTGCTAGCTTATCGACACACTGGCTGG	This study
P95	GCTAGCAAAGGAGAAGAACTCTT	This study
P96	TTATTTTTTGTTGATTACTTCTGGTAGCTT	This study
recA inner-F	TGARAARCARTTYGGTAAAGG	Wollenberg et al, 2012
recA inner-R	GGAGCRGCATCAGTCTCTGG	Wollenberg et al, 2012

[a]Restriction sites are underlined

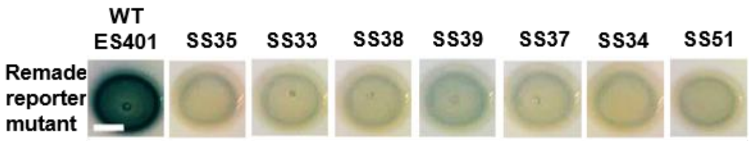

Figure S1. Reconstructed transposon mutants are unable to activate T6SS transcriptional reporter. Qualitative reporter activity for P_{hcp}-*lacZ* in wild-type ES401 and each transposon mutant reconstructed in an ES401 background. Scale bar is 2 mm. Images taken after 24 h on LBS-X-Gal. Assays were performed at least three times with four biological replicates and a representative experiment is shown.

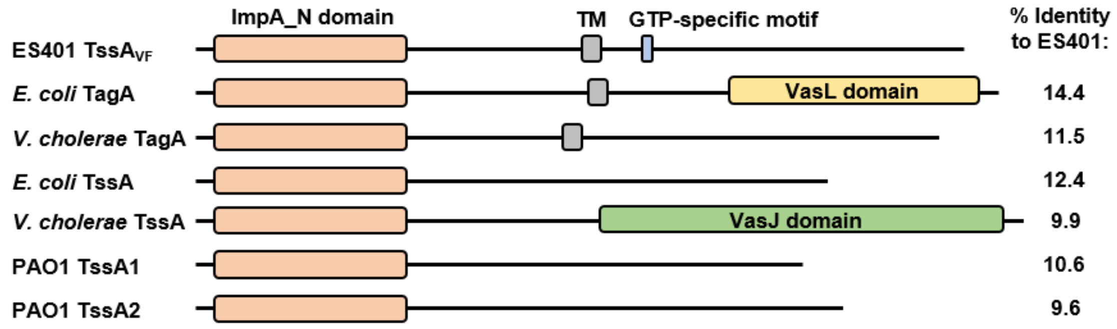

Figure S2. The *Vibrio fischeri* ImpA_N domain protein is distinct from TssA and TagA homologs. Domain architecture for *V. fischeri* ES401 $TssA_{VF}$ and ImpA_N domain proteins whose T6SS-related functions have been characterized in other species were identified using Hmmer. The GTP-binding motif (T/NKxD) was located by a manual sequence search and is only found in *V. fischeri* $TssA_{VF}$ and not found in homologs outside of the *V. fischeri* species. Percent identity to the ES401 protein was calculated by aligning the full-length proteins using Clustal Omega.

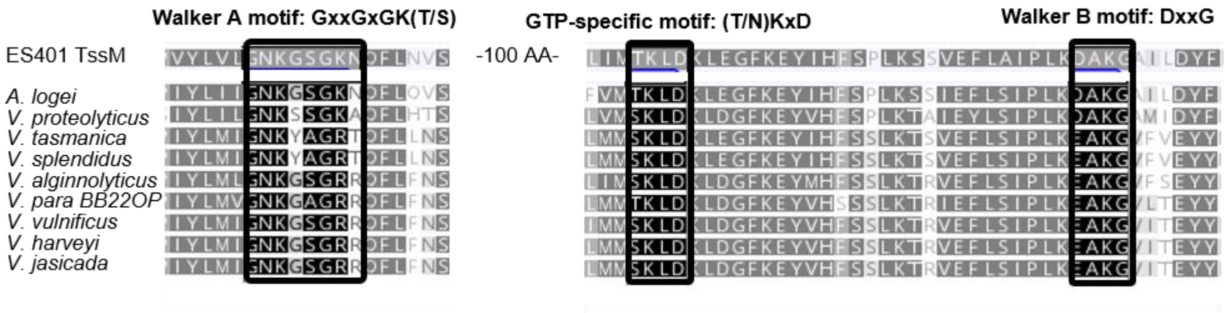

Figure S3. The *V. fischeri* TssM protein contains predicted GTPase domains. Walker A, Walker B, and GTP-specific binding motifs were identified by searching the ES401 TssM sequence manually. Analogous regions were identified by aligning the full-length sequences of ES401 TssM with closely-related vibrio homologs using Clustal Omega and visualized using Geneious. Black boxes outline motif regions and amino acid letter shading indicates conservation across species (black shading indicates a residue is 100% conserved).

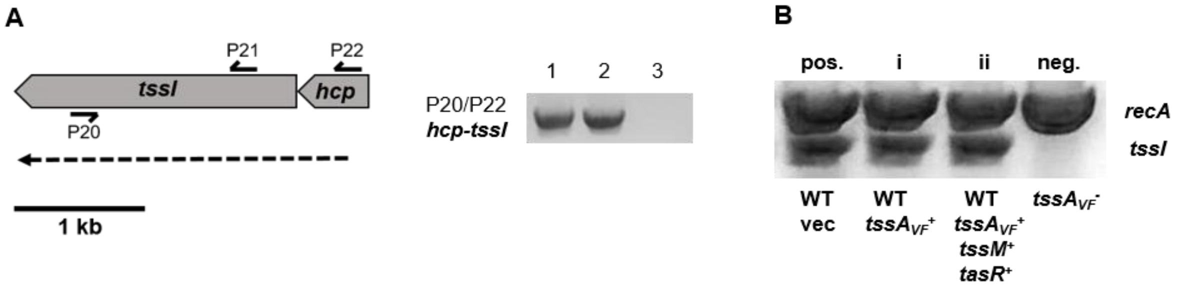

Figure S4. Co-transcriptional analysis of wild-type ES401 T6SS2 genes. (A) Graphic representation of *hcp* and *tssI* genes and the primers used for RT-PCR transcriptional analysis in wild-type ES401. The sequences for each primer can be found in Supplemental Table 5. Dashed arrows indicate predicted transcription based on RT-PCR results. Gel image depicting co-transcription of *hcp* and *tssI* in wild-type ES401 using primers P20 and P22. The lane assignments are as follows (1) wild-type ES401 chromosomal DNA as template; (2) wild-type ES401 RNA as template; (3) wild-type ES401 RNA as template, no reverse transcriptase. (B) Gel image based on RT-PCR results depicting relative abundance of *recA* and *tssI* transcripts in either wild-type ES401 carrying the empty vector (vec), a $tssA_{VF}$ expression vector, a $tssA_{VF}$-*tssM*-*tasR* expression vector, or the $tssA_{VF}^{-}$ mutant as a negative control. RNA was extracted from each strain following a 5 h incubation on LBS agar plates supplemented with 1.0 mM IPTG, and extractions and subsequent transcriptional analysis was performed twice.

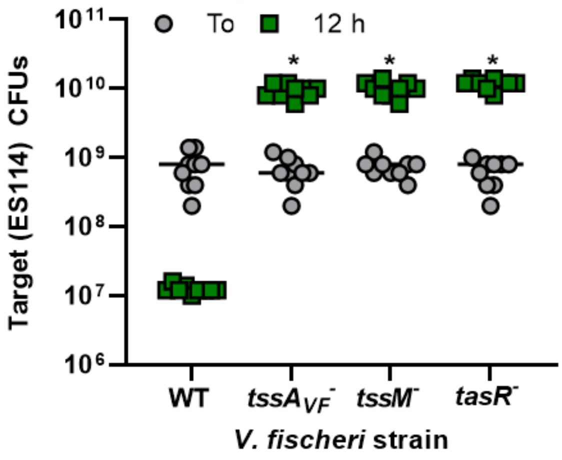

Figure S5. Tn mutants remain non-lethal in high-viscosity media. CFUs of ES114 at T0 (gray) or following a 12 h (green) coincubation with wild-type (WT) or mutant ES401 strains in a high-viscosity (hydrogel) liquid medium. *, $P<0.0001$ by a 2-way ANOVA followed by a Sidak's multiple comparisons test comparing 12 h CFUs from coincubation with each mutant to coincubation with WT ES401. Experiment was performed 3 times and combined data are shown (n = 12).
